# Quantitative evaluation of myocardial fibrosis by cardiac integrated backscatter analysis in Kawasaki disease

**DOI:** 10.1186/s12947-016-0046-7

**Published:** 2016-01-12

**Authors:** Lijian Xie, Renjian Wang, Min Huang, Yongwei Zhang, Jie Shen, Tingting Xiao

**Affiliations:** Department of Cardiology, Shanghai Children’s Hospital, Shanghai Jiaotong University, No. 355 Luding Road, Shanghai, 200062 China

**Keywords:** Kawasaki disease, Myocardial fibrosis, Cardiac integrated backscatter

## Abstract

**Background:**

Kawasaki disease is an acute, systemic vasculitis that affects the coronary arteries. However, the relationship between myocardial fibrosis and Kawasaki disease has been completely unknown until now. We aimed to provide quantitative information about myocardial fibrosis using cardiac integrated backscatter in Han race Kawasaki disease patients.

**Methods:**

Ninety Kawasaki disease patients and 90 healthy control subjects were recruited. Based on Kawasaki disease status, the patients were categorized into 3 groups: acute, subacute, and convalescence phase. Based on coronary artery status, the Kawasaki disease patients were categorized into 3 groups: without coronary artery lesions, with coronary artery dilation, and with coronary artery aneurysms. All subjects underwent two-dimensional and Doppler examinations to measure clinical echocardiographic parameters. Myocardial fibrosis was detected with calibrated integrated backscatter imaging.

**Results:**

Left ventricle systolic functions were normal in both the Kawasaki disease and control participants. The myocardial calibrated integrated backscatter values of the left ventricles of the acute (*p* < 0.001), subacute (*p* < 0.001) and convalescence phase (*p* < 0.001) Kawasaki disease patients were significantly greater than those of the healthy controls. The left ventricle myocardial calibrated integrated backscatter values were significantly smaller in the Kawasaki disease patients without coronary artery lesions than in the Kawasaki disease patients with coronary artery dilations and coronary artery aneurysms in different phases. The left ventricle myocardial calibrated integrated backscatter results were positively correlated with coronary artery status in the acute (*r* = 0.331, *p* < 0.001), subacute (*r* = 0.456, *p* < 0.001) and convalescence phases (*r* = 0.407, *p* < 0.001) of Kawasaki disease.

**Conclusion:**

Our findings may suggest that myocardial fibrosis occurs during early episodes of Kawasaki disease given uncertainties that exist regarding correlations of calibrated integrated backscatter and myocardial fibrosis.

## Background

Kawasaki disease is an acute, self-limited, systemic vasculitis of unknown etiology that most notably affects the coronary arteries [1]. The inflammatory process produces peculiar vascular damage that includes coronary artery lesions and myocarditis [2]. Myocardial ischemia remains an important concern, and regular evaluations of myocardial function constitute an important part of the long-term assessment of Kawasaki disease. Some authors have reported that Kawasaki disease patients might be at risk for developing ischemic cardiomyopathy due to abnormal myocardial perfusion and reduced coronary flow reserve, including patients who do not have coronary artery lesions [3–6].

Myocardial fibrosis in Kawasaki disease is still controversial due to limited evidence. Myocardial fibrosis has been documented in one patient via endomyocardial biopsy as long as 11 years after the occurrence of acute Kawasaki disease [7]. Lin et al. [8] found alterations in extracellular matrix biomarkers in Kawasaki disease patients that were suggestive of enhanced collagen synthesis and myocardial fibrosis in adolescents and young adults late after the onset of Kawasaki disease. With blood measurement and tissue autopsy, Numano et al. [9] found Galectin-3 could be a marker of myocardial and vascular fibrosis in Kawasaki disease patients with giant aneurysms. However, myocardial fibrosis was not observed in myocardial tissue of Kawasaki disease with normal coronary arteries [9].

However, myocardial biopsies are not practical for myocardial fibrosis diagnoses in children. Furthermore, the cardiac origin of circulating markers of collagen synthesis is difficult to ascertain, and the levels of these markers vary with age and growth, which may confound the interpretation of the results of pediatric studies [10]. In contrast, noninvasive assessment of tissue fibrosis via echocardiographically derived calibrated integrated backscatter has been used in both adults and children [11–13]. Moreover, calibrated integrated backscatter has been used to quantitatively evaluate the echogenicity of the heart in Kawasaki disease [13–16]. Recently, it is questionable about the role of calibrated integrated backscatter as a non-invasive index of fibrosis in clinical studies of patients without extensive fibrosis such as coronary artery disease [17].

However, data derived from small samples, specificities of the equipment used, different measurements of calibrated integrated backscatter and variability due to race may have important effects on calibrated integrated backscatter data. Therefore, the reproducibility of calibrated integrated backscatter measurements needs to be examined in large samples to confirm the reported results. Moreover, whether calibrated integrated backscatter is associated with lesser degrees of fibrosis is unknown. In the present study, we postulated that calibrated integrated backscatter might provide quantitative information about myocardial fibrosis in early episode of Kawasaki disease. Furthermore, we explored the associations of fibrosis with myocardial calibrated integrated backscatter results and the statuses of coronary artery lesions in Kawasaki disease patients.

## Methods

### Subjects

Ninety Kawasaki disease patients and 90 healthy controls were prospectively recruited from a group of cardiac in-patients and from the clinic (from January 2014 to January 2015). The healthy controls underwent echocardiography examinations for soft heart murmurs in the clinic, and heart disease was completely ruled out for these subjects. The following data were collected from the case notes: demographic data, age at diagnosis, development of coronary artery complications, and current cardiac medications. Based on Kawasaki disease status, the patients were categorized into the following 3 groups: group 1, acute phase (5-10^th^ day); group 2, subacute phase (11-21^th^ day), and group 3, convalescence phase (28^th^ day – 3^rd^ month). Group 4 was composed of the healthy controls. Based on coronary artery status, the Kawasaki disease patients were categorized into the following 3 groups: group A, no coronary artery lesions; group B, coronary artery dilation; and group C, coronary artery aneurysms. Group D was composed of the healthy controls. The echocardiographic data were compared between the patients and the healthy age-matched control subjects.

Diagnoses of Kawasaki disease were made based on standard clinical criteria [18]. Coronary artery dilations and coronary artery aneurysms were documented by echocardiography according to the criteria set by the Japanese Ministry of Health [19].

### Ethics statement

This study was approved by the Institutional Review Board of Shanghai Children’s Hospital/Shanghai Jiaotong University, and informed written consent was obtained from all subjects and parents on behalf of minors.

### Clinical echocardiography

Echocardiography was performed by a single experienced pediatric cardiologist. All the Kawasaki disease patients and controls underwent transthoracic two-dimensional and Doppler examinations using a Philips IE-33 ultrasound machine. The probes used were S4 and S8. The children were prepared for echocardiography via the administration of a sedative (10 % chloral hydrate 50 mg/kg administered orally) as usually required in clinical practice in China.

### Calibrated integrated backscatter assessment

**Fig. 1:**
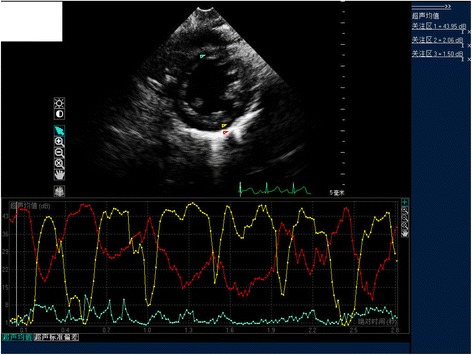
Calibrated integrated backscatter (cIB) of the left ventricle (LV) assessed with QLAB software

The calibrated integrated backscatter was assessed using QLAB software. The calibrated integrated backscatter measurements were performed as previously reported [11]. Integrated backscatter curves were acquired in the parasternal short-axis view by locating 3 × 3-mm sample volumes in the mid-basal septum, mid-posterior wall, and mid-pericardium in end diastole. The calibrated integrated backscatter measurements were performed at a fixed point in the cardiac cycle (i.e., the peak of the QRS complex) and are expressed in decibels. The pericardial integrated backscatter was used as the baseline. The left ventricular calibrated integrated backscatter was calculated by subtracting the average pericardial integrated backscatter intensity from the average myocardial integrated backscatter intensity of the septum and posterior wall (Fig. 1). The sample volumes were tracked manually to maintain the same region throughout the heart cycle.

### Reproducibility

Off-line analyses of the calibrated integrated backscatter data were performed by two blinded independent observers. Interobserver variability was tested in 10 cases of Kawasaki disease and 10 control cases. The same samples were re-evaluated after a 20-day period by one of the two observers to assess intraobserver variability.

### Statistical analysis

All clinical echocardiographic data and integrated backscatter profiles were averaged over three consecutive cardiac cycles. The data are presented as the means ± the SDs unless otherwise stated. The demographic and echocardiographic parameters were compared between the patients with different Kawasaki disease statuses and the controls using unpaired Student's t tests. The calibrated integrated backscatter values of the patients in the different phases of Kawasaki disease and the healthy controls were compared with one-way ANOVA tests. Tukey’s post-hoc test was used whenever statistically significant differences were observed. Spearman correlation analysis was used to determine the association between the myocardial calibrated integrated backscatter results and the coronary artery statuses of the Kawasaki disease patients. P values <0.05 were considered statistically significant. All statistical analyses were performed using SPSS version 16.0.

## Results

### Subjects

All subjects were of Han nationality. The 90 (58 males, 64.4 %) Kawasaki disease patients were studied at 18.40 ± 10.07 months, and the 90 (55 males, 61.1 %) healthy controls were studied at 17.93 ± 11.59 months (*p* = 0.773). Neither gender (64.4 % vs. 61.1 %, *p* = 0.646) nor body weight (11.72 ± 3.03 kg vs. 11.41 ± 3.48 kg, *p* = 0.525) differed significantly between the Kawasaki disease and control cohorts.

The gender distribution was different (47 males, 62.7 % vs. 10 males, 83.3 % vs. 1 male, 33.3 % vs. 55 males, 61.1 %, *p* < 0.001) in subgroup A, B, C and D. Neither age (17.87 ± 9.74 vs. 21.92 ± 11.83 vs. 17.67 ± 12.22 vs. 17.93 ± 11.59 months, *p* = 0.675) nor body weight (11.63 ± 2.91 kg vs. 12.28 ± 3.70 kg vs. 11.57 ± 4.05 kg vs. 11.41 ± 3.48 kg, *p* = 0.846) differed significantly in subgroup A, B, C and D.

All patients were treated with 2 g/kg immunoglobulin intravenously in a single dose and 50–80 mg/kg aspirin during the acute phase of the illness (5-10^th^ day) followed by 3–5 mg/kg per day for 6–8 weeks after the resolution of the acute illness. Aspirin was continued chronically at the same dose in children in whom coronary abnormalities persisted.

### Clinical echocardiographic parameters

Table 1 summarizes the clinical echocardiographic parameters in patients in the different phases of Kawasaki disease and the control subjects. Left ventricle systolic and diastolic functions were normal, and no significant differences in either parameter were observed between the Kawasaki disease patients and controls. Left ventricle masses did not differ across all of the subjects. Among the Kawasaki disease cohorts, 75 (83.3 %) cases were without coronary artery lesions, 12 (13.3 %) cases exhibited coronary artery dilation, and 3 (3.3 %) cases had coronary artery aneurysms.

**Table 1:** Clinical echocardiographic parameters in KD and control

	KD (acute)	KD (subacute)	KD (convalescence)	Healthy control	*p*
*Left Ventricle*					
*M-mode*					
LV EDD (mm)	33.6 ± 3.4	32.9 ± 3.6	33.3 ± 3.2	32.7 ± 2.5	0.56
LV ESD (mm)	17.1 ± 1.9	17.6 ± 1.4	16.9 ± 2.0	17.2 ± 1.7	0.32
IVSd (mm)	4.2 ± 0.5	4.3 ± 0.3	4.1 ± 0.4	4.1 ± 0.3	0.38
LV PWd (mm)	4.0 ± 0.4	4.1 ± 0.4	4.0 ± 0.5	4.1 ± 0.4	0.62
LV mass (g)	22.9 ± 0.4	22.3 ± 0.5	22.8 ± 0.5	23.0 ± 0.4	0.33
LV eject fraction (%)	68.3 ± 4.2	70.2 ± 6.1	69.5 ± 5.7	68.9 ± 5.5	0.14
*Mitral inflow velocities*					
E (cm/s)	97.7 ± 10.2	105.4 ± 9.7	93.8 ± 13.5	98.7 ± 12.5	0.49
A (cm/s)	59.9 ± 12.5	57.5 ± 14.7	61.4 ± 13.2	62.3 ± 11.9	0.78
E/A ratio	1.6 ± 0.4	1.8 ± 0.4	1.5 ± 0.3	1.6 ± 0.4	0.65
*Right ventricle*					
*Tricuspid inflow velocities*					
E (cm/s)	71.0 ± 12.1	74.3 ± 10.5	69.8 ± 8.4	73.4 ± 15.1	0.84
A (cm/s)	50.0 ± 6.9	54.9 ± 9.8	53.8 ± 7.3	51.5 ± 5.3	0.33
E/A ratio	1.4 ± 0.3	1.4 ± 0.2	1.3 ± 0.2	1.4 ± 0.3	0.79

*Abbreviations*: *KD* Kawasaki disease, *LV* left ventricle, *EDD* end-diastolic dimension, *ESD* end-systolic dimension, *IVSd* interventricular septal thickness at diastole, *PWd* posterior wall thickness at diastole

### Myocardial calibrated integrated backscatter

**Fig. 2:**
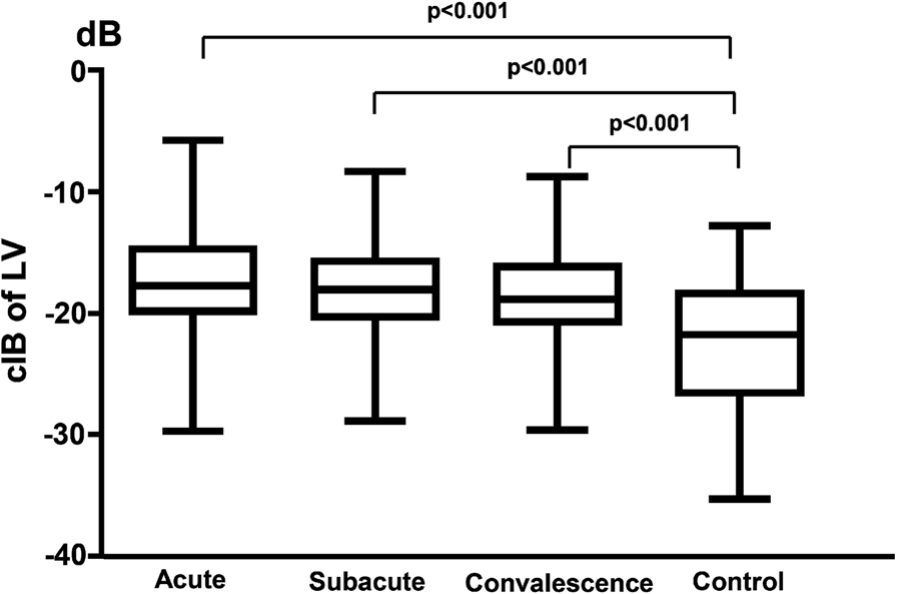
Bar graph illustrating the calibrated integrated backscatter (cIB) values of the left ventricles (LVs) in different phases of KD and in healthy control subjects

**Fig. 3:**
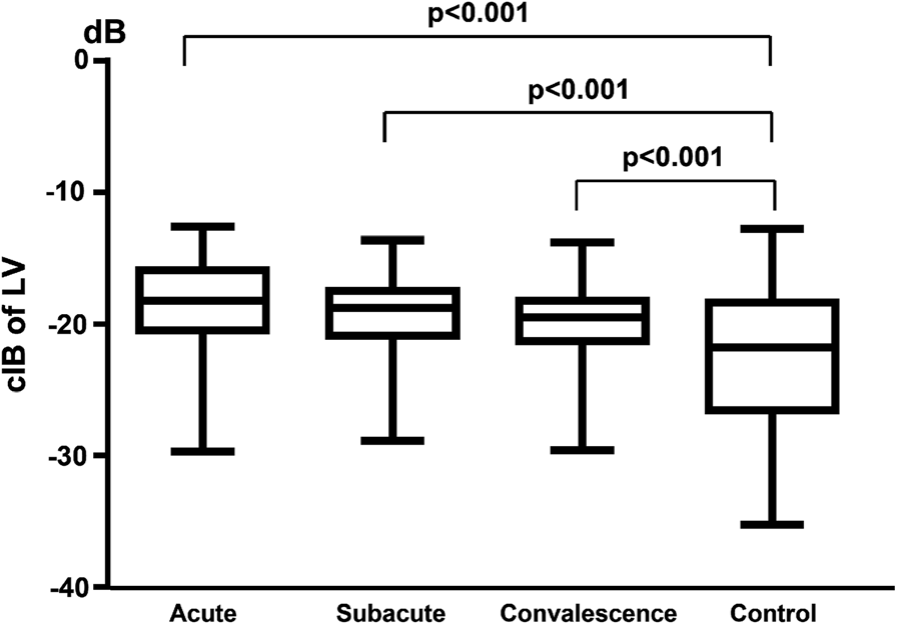
Bar graph illustrating the calibrated integrated backscatter (cIB) values of the left ventricles (LVs) in different phases of KD without coronary artery lesions (CALs) and in healthy control subjects

**Fig. 4:**
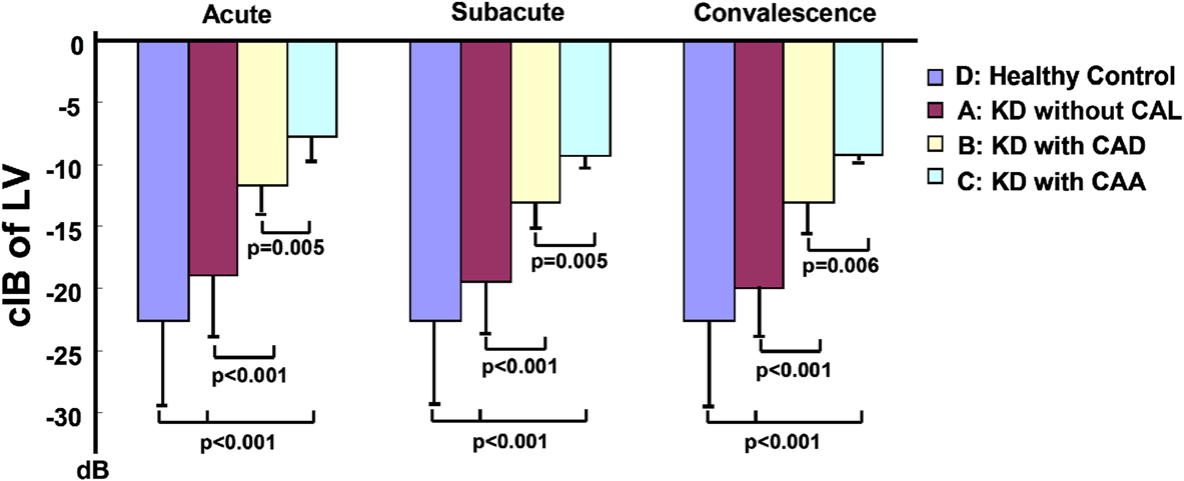
Histogram illustrating the calibrated integrated backscatter (cIB) values for the left ventricles (LV) in the different KD phase cohorts with and without coronary artery lesions (CALs). CAD: coronary artery dilation; CAA: coronary artery aneurysm

The myocardial calibrated integrated backscatter values for the left ventricle were significantly smaller in the healthy control subjects than the Kawasaki disease patients in the acute (−22.57 ± 5.43 vs. -17.60 ± 4.78, *p* < 0.001), subacute (−22.57 ± 5.43 vs. -18.31 ± 4.27, *p* < 0.001) and convalescence (−22.57 ± 5.43 vs. -18.71 ± 4.30, *p* < 0.001) phases (Fig. 2). Furthermore, the myocardial calibrated integrated backscatter values for the left ventricle were significantly smaller in the healthy controls than in the Kawasaki disease patients without coronary artery lesions in the acute (−22.57 ± 5.43 vs. -18.93 ± 3.95, *p* < 0.001), subacute (−22.57 ± 5.43 vs. -19.51 ± 3.50, *p* < 0.001) and convalescence (−22.57 ± 5.43 vs. -19.98 ± 3.36, *p* < 0.001) phases (Fig. 3). Finally, the myocardial calibrated integrated backscatter values for the left ventricle were significantly greater in the Kawasaki disease patients with coronary artery aneurysms and coronary artery dilation compared with the Kawasaki disease patients without coronary artery lesions in the acute (−7.77 ± 1.71 vs. -11.71 ± 1.85 vs. -18.93 ± 3.95), subacute (−9.27 ± 0.85 vs. -13.08 ± 1.85 vs. -19.51 ± 3.5) and convalescence phase (−9.23 ± 0.31 vs. -13.13 ± 2.02 vs. -19.98 ± 3.36) of illness (Fig. 4).

### Correlates of calibrated integrated backscatter

**Fig. 5:**
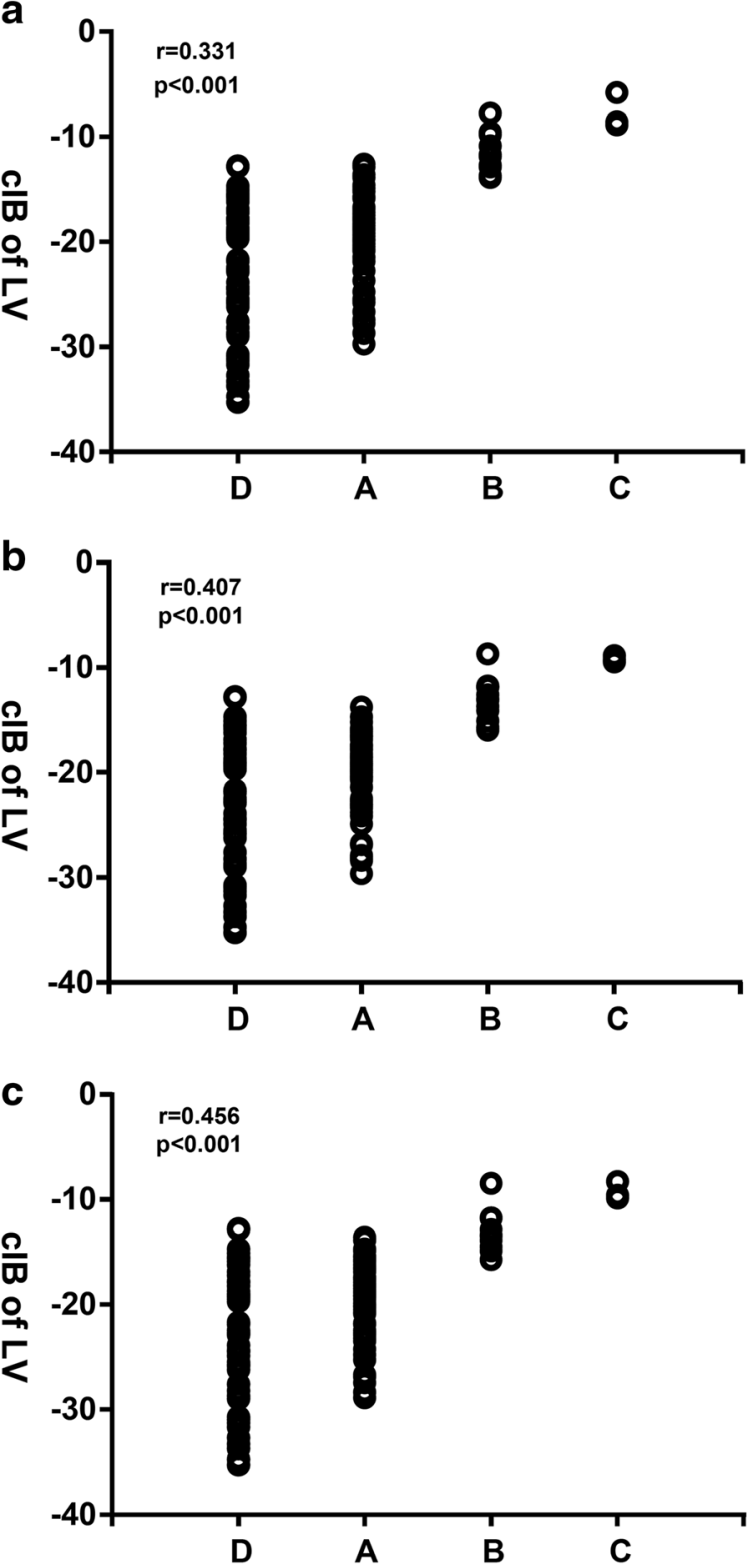
Scatter plots illustrating the correlations between the myocardial calibrated integrated backscatter values and coronary artery statuses in (**a**) the acute phase, (**b**) the subacute phase, and (**c**) the convalescence phase. D: healthy controls; A: KD without CAL; B: KD with CAD; C: KD with CAA

Among the cohort of patients, the average myocardial calibrated integrated backscatter values were positively correlated with the Kawasaki disease patients’ coronary artery statuses in the acute (*r* = 0.331, *p* < 0.001), subacute (*r* = 0.456, *p* < 0.001) and convalescence (*r* = 0.407, *p* < 0.001) phases (Fig. 5).

### Reproducibility

The intra- and interobserver variability for the calibrated integrated backscatter observations was 1.6 ± 1.5 (correlation was 0.89 (95 % CI 0.75 to 0.95, *p* < 0.001)), and 1.7 ± 1.3 (correlation was 0.83 (95 % CI 0.70 to 0.89, *p* < 0.001)), respectively.

## Discussion

The present study demonstrated that increased myocardial calibrated integrated backscatter values are suggestive of myocardial fibrosis in Kawasaki disease patients with and without coronary artery lesions despite similar measures of left ventricle function between the Kawasaki disease patients and healthy controls. Additionally, the myocardial calibrated integrated backscatter results were found to be positively associated with the Kawasaki disease patients’ coronary artery statuses.

Calibrated integrated backscatter is the intensity of an ultrasound wave (scattering) within a particular frequency range, as reflected by a tissue interface that is smaller than the ultrasound wavelength (500 μm for a 3-MHz probe); thus, the structures that contribute to the calibrated integrated backscatter signal should be 100–125 μm or smaller [20]. Myofibers, extracellular matrix collagen, mitochondria, and small vessels are thought to be the principal backscatterers in the myocardium [21]. With the development of echocardiolographic technologies, the relationship between calibrated integrated backscatter and myocardial fibrosis has been confirmed in human disease [12, 22–24].

Kawasaki disease is a systemic vasculitis of unknown origin that occurs primarily in children younger than 5 years. Many studies have focused on Kawasaki disease with coronary artery lesions. However, few pathological studies have examined the after-effects of Kawasaki disease on the myocardium based on endomyocardial biopsies [7, 25]. Recently, Yonesaka et al. [26] found that the incidence of histopathological abnormalities, such as degeneration, hypertrophy, and inflammatory cell infiltration, are quite high upon initial study and that inflammatory cell infiltration, interstitial fibrosis, and disarray were very noticeable upon follow-up biopsies in Kawasaki disease with giant coronary artery aneurysm. Furthermore, a previous clinical study provided evidence of altered myocardial extracellular matrix turnover with a possible predisposition to increased interstitial fibrosis in adults with Kawasaki disease histories [8]. Leonardi et al. [13] confirmed a significant increase in myocardial calibrated integrated backscatter values in Kawasaki disease patients at least 1 year after the acute episode. Nagata et al. [14] reported that the integrated backscatter values of the coronary artery wall, mitral valve, papillary muscle and ascending aortic wall in Kawasaki disease patients in the acute phase are significantly higher than those of control subjects. Abe et al. [15] also reported that the integrated backscatter values for the coronary artery wall are higher in Kawasaki disease patients than in controls. Moreover, these authors also reported that the integrated backscatter values for the coronary artery wall are higher in pre-immunoglobulin therapy patients than post-immunoglobulin therapy patients with Kawasaki disease. However, Yu et al. [16] used a similar method and reported no significant difference in the perivascular echo brightness of the coronary arteries between Kawasaki disease patients.

The numbers of Kawasaki disease and control subjects are much greater in the present study than those in previous reports. However, the present study is the first to report on the echogenicity of the left ventricle myocardium in Han race Kawasaki disease patients based on the calibrated integrated backscatter method with QLAB software. Our results demonstrated greater myocardial calibrated integrated backscatter values in the Kawasaki disease patients in the acute, subacute and convalescence phases compared with controls. Moreover, greater myocardial calibrated integrated backscatter values observed in the Kawasaki disease patients with coronary artery lesions compared with the Kawasaki disease patients without coronary artery lesions. Finally, we revealed that the calibrated integrated backscatter values were positively correlated with the Kawasaki disease patients’ coronary artery statuses.

Calibrated integrated backscatter data have been correlated with myocardial collagen distribution and content and are thought to be a marker of fibrosis [12, 13]. Myocarditis is uniformly present during the acute phase of Kawasaki disease and resolves early in its clinical course. An increase in collagen content is a common response to myocarditis and might have been the cause of the elevated calibrated integrated backscatter values observed in our patients. Thus, histological alterations, such as edema and cell infiltration associated with inflammation of tissues, alter the acoustic properties of tissues [27]. The results of the present study suggest that edematous changes and cell infiltration in the ventricular myocardium will develop in the acute phase of Kawasaki disease and persist for a long time. Our data indicate that myocardial fibrosis may occur during early episodes of Kawasaki disease and that calibrated integrated backscatter is a simple, easy, and noninvasive echocardiographic method for the evaluation of myocardial fibrosis in Kawasaki disease.

Several limitations to this study warrant discussion. First, the subjects’ thoracic thicknesses may have affected the calibrated integrated backscatter values, but we did not consider this issue. Second, the modality of cardiac magnetic resonance imaging (MRI) T1 mapping, which is ideal for assessments of diffuse myocardial fibrosis, was not available to us due to economic reasons and the requirement for deeper sedation. Notably, however, conditions other than fibrosis may also influence extracellular volume measurements acquired via T1 mapping [28]. Third, we did not examine circulating markers of collagen synthesis in the present study. The cardiac origins of these collagen synthesis biomarkers are difficult to ascertain. More importantly, the levels of these markers vary with age and growth, which may confound the interpretation of the results of pediatric studies [10]. Fourth, lack of histological evidence, we could not evaluate the extensive myocardial fibrosis in Kawasaki disease. So, calibrated integrated backscatter maybe reflect not only fibrosis but also myocardium inflammation especially in acute phase. Finally, we did not examine the LV strains or strain rates with tissue Doppler or two-dimensional echocardiography in the present study.

## Conclusion

Our findings suggest that calibrated integrated backscatter values are different in Kawasaki disease and may suggest myocardial fibrosis occurs during early episodes of Kawasaki disease given uncertainties that exist regarding correlations of calibrated integrated backscatter and myocardial fibrosis. Furthermore, calibrated integrated backscatter value is related to the status of the coronary artery in Kawasaki disease. Our findings reinforce the concept that long-term follow-up is appropriate for all Kawasaki disease patients independent of the presence of coronary artery lesions. Whether anti-fibrotic strategies, such as aldosterone antagonism, may alter myocardial acoustic properties and improve myocardial fibrosis progression in Kawasaki disease is a topic for further studies.
